# Effects of low-dose contraceptive pills on the risk factors of cardiovascular diseases among 15-35-year-old women: A retrospective cohort

**DOI:** 10.18502/ijrm.v17i10.5496

**Published:** 2019-11-28

**Authors:** Zahra Momeni1comma, Ali Dehghani, Ph.D., Hossein Fallahzadeh, Moslem Koohgardi3 M.Sc. Student, Maryam Dafei, Masoud Mohammadi

**Affiliations:** ^1^Deputy of Health, Bushehr University of Medical Sciences, Bushehr, Iran.; ^2^Department of Biostatistics and Epidemiology, Faculty of Health, Shahid Sadoughi University of Medical Sciences and Health Services, Yazd, Iran.; ^3^Department of Health Education and Health Promotion, Faculty of Health, Rafsanjan University of Medical Sciences and Health Services, Kerman, Iran.; ^4^Department of Midwifery, School of Nursing and Midwifery, Shahid Sadoughi University of Medical Sciences and Health Services, Yazd, Iran.; ^5^Department of Nursing, Faculty of Nursing and Midwifery, Kermanshah University of Medical Sciences, Kermanshah, Iran.

**Keywords:** Birth control pills, Homocysteine, Nitric oxide, Dyslipidemias, Coronary artery diseases.

## Abstract

**Background:**

Cardiovascular diseases could be preventable; as a result, understanding the risk factors was regarded as the major priority for healthcare providers.

**Objective:**

The main objective of this research was to achieve a deeper insight into the effect of long-term use of low-dose oral contraceptive pills (OCP) on the risk factors of cardiovascular diseases.

**Materials and Methods:**

This research was a retrospective cohort conducted (historical and prospectively) conducted on 100 women with normal menstrual cycles aged 15 to 35 yr, who were referred to the healthcare centers in Yazd, Iran. The participants were categorized into two groups: The OCP group was consuming the pills for 0-3, 4-23, and 24-36 months, and the non-OCP group. Participants were followed up for a minimum of 3 months and a maximum of six months.

**Results:**

The highest level of low-density lipoprotein (LDL), homocysteine, cholesterol, triglyceride levels, and systolic blood pressure was observed in the OCP group in the duration of 24 to 36 months. The Tukey's test demonstrated that there were comprehensible differences in the LDL (p = 0.01), cholesterol (p = 0.01), triglyceride (p < 0.001), and homocysteine levels (p < 0.001), also systolic blood pressure (p = 0.04).

**Conclusion:**

It was realized that the long-term consumption of low-dose OCP can augment the incidence of some risk factors (systolic blood pressure, homocysteine levels, cholesterol, LDL-c, and triglyceride) and lead to developing cardiovascular diseases amongst the healthy women.

## 1. Introduction

Cardiovascular diseases are predicted to remain as the most significant contributing factor for mortality (26% of total deaths) until 2020 in all over the world. Furthermore, they are supposed to be the leading cause of mortality in Iran (1). Women in reproductive age are less prone to cardiovascular diseases compared to men of the same age (2). Although OCPs have been used worldwide as the most popular method to prevent pregnancy for more than half a century (2, 3), researchers have demonstrated that there is a relationship between combined hormonal OCPs and hypertension (2, 4). Hyperhomocysteine increased plasma homocysteine (HCY) concentration (5), so it has been reported as a cardiovascular disease risk factor over the last decade (6, 7). The HCY level in premenopausal women is 20% lower than in men, however fasting HCY level increases after menopause and reaches the level in men (8, 9).

HCY is a sulfur-contained amino acid and as a by-product of methionine breakdown (10) prevents the normal function of nitric oxide (NO) resulting from the autoxidation process. It can, therefore, cause endothelial dysfunction (11). NO is produced in the endothelium by L-arginine synthesis (11) and can cause vasodilatation, -preventing platelet accumulation- and migration (12, 13). In a study it was shown that a decrease in NO may lead to diseases like atherosclerosis, cardiac disease, and hypertension (14).

Various studies have demonstrated that female gonadal steroids may affect NO and HCY (12, 13). However, because of the contradictory results, it is still unclear whether an oral contraceptive pill (OCP) is related to changes in plasma HCY levels (15, 16) or not. Nowadays, low-dose pills containing 0.15 mg of levonorgestrel and 0.03 mg of ethinylestradiol (16) are considered as the usage contraindication of the pills after the age of 35 yr. Nevertheless, the pills were widely administered on women who are nonsmoker and without hypertension and migraine, which predisposes them to cardiovascular disease risk (17, 18).

Furthermore, it has been reported that OCPs can change some plasma lipids concentration including total cholesterol, high-density lipoprotein cholesterol (HDL-c), low-density lipoprotein cholesterol (LDL-c), and triglyceride (15-18). Consuming OCPs can have a negative effect on the lipid profiles in healthy women, that is, it can increase the triglyceride level and also decrease the high-density lipoproteins (HDL) level (18).

Although consuming OCPs can increase cardiovascular risk factors, there are limited reports in this regard in the literature. Therefore, this research is designed and conducted to investigate the relationship between cardiovascular disease risk factors and the consumption of low-dose OCPs amongst healthy women in Yazd, Iran.

## 2. Materials and Methods 

This survey was a retrospective cohort, conducted on 100 married women with normal menstrual cycles aged 15 to 35 yr referred to the healthcare centers and also family planning clinics including Rahmht Abad, Azadshahr, Akbari, and Dehno Health Center in Yazd, Iran.

The sample population was followed-up in 2014 for a minimum duration of three months and a maximum of six months and the OCP users' blood samples were examined for two years.

### Subjects

Participants were classified into OCP and non-OCP groups. The OCP group consisted of women with menstrual cycles of at least three days and a maximum of 36 days, who used OCP (Aburaihan Pharmaceutical Co., Iran) for 0-3, 4-23, and 36- 24 months. After taking 21 pills, they stopped taking the pills for 7 days, then they took the next box of pills. The non-OCP group included women referred to the health care centers for any other reasons and did not take any birth birth-control hormonal preparations.

### Sample size and sampling method

The significance level of 5% and the test power of 90% were used for this study. Considering the standard deviation of HCY level in the previous study (SD = 3.5) (19), for achieving a minimum difference of more than 3 units between the mean HCY levels after a maximum of six months follow-up in both groups, according to the following formula, 50 samples per group were required: 

$$n=\frac{{\left(Z_{\frac{1-\alpha }{2}}+Z_{\beta }\right)}^{2}2S^{2}}{{\left({\underline{x}}_{1}-{\underline{x}}_{2}\right)}^{2}}.$$

### Data collection

The demographic data including the age, occupation, educational level, family income level, as well as data regarding the history of pregnancy, delivery type, number of children, smoking and alcohol habits, history of taking medication (e.g., OCPs), menstrual cycle, duration of OCP usage, and physical exercise were obtained through a face-to-face interview. The exclusion criteria included repeated abortion history (≥ 2 times), thyroid dysfunction, history of heart diseases in family before the age of 40 yr, personal history of heart disease, diabetes, hepatic disease, renal dysfunction, and dyslipidemia. Other exclusion criteria encompassed those working with pesticides, smoking themselves or having a partner who smokes, drinking alcohol, history of anemia, taking vitamin supplements such as folic acid, vitamins B6 and B12 during the last year, receiving a blood transfusion, and being pregnant since the last year. The essential information was gathered through face-to-face interviews and recorded on a checklist.

Also, a literature survey was done to determine the factors influencing lipid profiles, HCY, and NO levels (9-18).

### Procedures

After consulting specialists, some factors influencing blood biochemical parameters were determined and recorded and ultimately prepared as a checklist validated by authorities. Based on this checklist, the candidates who were not meeting the exclusion criteria were included in the study. The participants were categorized into four groups. The first group had not consumed those pills or using any other hormonal method to prevent pregnancy. Groups 2, 3, and 4, on the other hand, had consumed LD tablets for a time period of 3, 4-23, and 24-36 months, respectively.

The participants were followed-up for a minimum duration of three months and a maximum of six months. After that, their weight, blood pressure, body mass index (BMI) (manufactured by OMRON health care BF511, China), and waist-hip ratio (WHR) were measured. In the end, the venous blood samples were taken from the groups (OCP and non-OCP groups) and were sent to the central laboratory in Yazd to analyze the biochemical parameters using the standard techniques (2, 17, 20-23).

### The conditions and methods of blood biochemical parameter measurements

10cc blood sample was taken from each participants between 9 and 11 am to minimize the chemical changes. 4 ml was put into EDTA tubes, and 6 ml in the sodium citrate tubes being kept in the ice throughout the preparation process, during the time of the sampling phase until the samples were sent to the central laboratory for analyzing the parameters. Total cholesterol, triglyceride, HDL-c, and LDL-c levels were measured. The following parameters were calculated using auto Analyzer (Hitachi, Japan) and commercial kits, manufactured by Bionic corporation (confirmed by the Iran Health Reference Laboratory): triglyceride levels in serum by using the enzymatic method (lipase for converting triglyceride to glycerol), cholesterol levels by using the enzymatic cholesterol esterase (lipase for converting triglyceride to glycerol), and HDL-c by using the enzymatic precipitation method. LDL-c was also calculated using the Friedwald formula (24).

HCY levels were determined (coefficient of variation ≤ 5%) via the enzymatic and photometric method using the HCY kit manufactured by Diazyme (Roche subsidiary company, USA) and Auto Analyzer BT3000 (Biotechnics, Italy). The NO levels were determined using a photometric method and also Greiss reaction. A micropipette reader (having CV < 5%), a kit manufactured by Iran Sib Bio, and an ELISA microplate reader 3200 FaxStat (Awareness Technologies, USA) were used in this work.

### Ethical consideration

This study was approved by Shahid Sadoughi University of Medical Sciences Ethics Committee, Yazd, Iran (Code: IR.SSU.SPH.REC.1394.69). Written informed consent was obtained from all participants in this study.

### Data analysis

The collected data were analyzed using the Statistical Package for the Social Sciences software (SPSS, version 16, SPSS Inc, Chicago, Illinois, USA), the descriptive tests, Chi-square test, independent *t*-test, Tukey's test, and ANOVA test with a significance level of 0.05. In conclusion, the results were attained by comparing the participants consuming the pills with those using non-hormonal birth control methods.

## 3. Results

In this study, 100 women aged 15-35-yr-old were investigated. The basic characteristics of the participants are displayed in Table I. In this study, two groups were matched for age (Table I). The women consuming the pills for the least amount of time had the highest rate of pregnancy.

According to the results, a majority of participants in the OCP group were housewives that is 88.8%, 94.4%, and 91.3% of those exposed to the pills for 3, 4-23, and 24-36 months, respectively. Moreover, regarding the education level of the participants, in the groups exposed to the pills for 3 and 24-36 months, a majority of the participants had primary school education degree or were illiterate. On the contrary, in the non-OCP group, most of the participants were housewives with a university degree (Table I).

Based on our findings, there were no comprehensible statistical differences regarding job (p = 0.525), educational level (p = 0.636), and hourly physical exercises in a week (p = 0.830). Although there was no significant difference statistically between the two groups, there was a comprehensible statistical association between the groups concerning the monthly family income level (p = 0.011).

Anthropometric features and the participants' blood pressure measurements are shown in Table II. Using ANOVA test, no statistically significant differences were found in the mean values of BMI, WHR, and diastolic blood pressure amongst the groups regarding the duration of the consumption of the pills (p ≥ 0.05), while there were comprehensible differences in the systolic blood pressure amongst the groups regarding the pills-usage duration. Tukey's test proved that the maximum mean systolic blood pressure was seen amongst the women consuming the pills for 4-23 and 24-36 months (p = 0.05).

The average incidence rates of risk factors for increasing cardiovascular diseases amongst the participants are displayed in Table III. There were no remarkable differences in the mean values of the NO and HDL levels amongst the groups regarding the duration of the consumption of LD contraceptive pills according to the ANOVA test. However, there were comprehensible differences in the mean values of LDL, HCY, cholesterol, and triglyceride levels between the groups due to the difference in the duration of the consumption of LD contraceptive pills statistically. Tukey's test proved that the mean values of LDL (p = 0.02), cholesterol (p = 0.01), and triglyceride (p < 0.001) levels amongst the users of the pills for 24-36 months and the mean value of HCY level among the users for 4-23 and 24-36 months were higher than the mean values of those levels amongst the other corresponding person (p < 0.001).

**Table 1:** The basic participants' characteristics (n = 100) based on the duration of the consumption of LD contraceptive pills


**Variables**	**The time of consuming LD contraceptive pills**				**P-value**
	**Not consuming the pills**	**3 Months**	**4-23 Months**	**24-36 Months**	
The number of participants	50	9	18	23	
Age*	30.06 ± 4.06	31.11 ± 3.55	27.55 ± 4.46	31.73 ± 2.98	<brow>-2</erow> 0.322a
Job**					
Housekeeper	43 (86)	8 (88.8)	17 (94.4)	21 (91.3)	
Employee	7 (14)	1 (11.2)	1 (5.6)	2 (8.7)	<brow>-2</erow> 0.525b
Education level**					
Illiterate + elementary school	9 (18)	4 (44.4)	3 (16.7)	7 (30.5)	
Middle school	11 (22)	0 (0.0)	5 (27.7)	5 (21.7)	
Diploma	14 (28)	4 (44.4)	4 (22.3)	6 (26.1)	
Upper diploma	16 (32)	1 (11.2)	6 (33.3)	5 (21.7)	<brow>-4</erow> 0.636b
The numbers of pregnancy*	2.08 ± 0.98	2.33 ± 0.86	2.22 ± 1.06	2.26 ± 0.54	0.201a
Doing physical exercises**					
Never	31 (62)	6 (66.7)	10 (55.5)	12 (52.2)	
Less than 2 hr per week	12 (24)	0 (0.0)	5 (27.8)	9 (39.2)	
≥ 3 hr per week	7 (14)	3 (33.3)	3 (16.7)	2 (8.6)	<brow>-3</erow> 0.830b
Income per month${}^{**}$					
< 5,000,000 R	1 (2)	2 (22.2)	3 (16.6)	3 (13.1)	
5,000,000-10,000,000 R	33 (66)	6 (66.6)	11 (61.2)	18 (78.2)	
> 10,000,000R	16 (32)	1 (11.2)	4 (22.2)	2 (8.7)	<brow>-3</erow> 0.011b
*Data presented as n (%), ** Data presented as Mean ± SD					
${}^{a}$ Independent student's *t*-test; ${}^{b}$ ANOVA test

**Table 2:** The mean values and SD of anthropometric characteristic indexes and participants' blood pressure measurements in terms of the consumption of LD contraceptive pills duration


**Anthropometric indexes and blood pressure measurements**	**The duration of consuming LD contraceptive pills (month)**	**Numbers**	**Mean** ± ** SD**	P-value*
	Not consuming the pills	50	25.35 ± 4.85	
	3 months	9	26.66 ± 3.87	
	4-23 months	18	24.78 ± 4.67	
<brow>-4</erow> BMI(Kg/m${}^{2}$)	24-36 months	23	26.74 ± 3.19	<brow>-4</erow> 0.42
	Not consuming the pills	50	0.85 ± 0.07	
	3 months	9	0.84 ± 0.05	
	4-23 months	18	0.83 ± 0.07	
<brow>-4</erow> WHR	24-36 months	23	0.85 ± 0.06	<brow>-4</erow> 0.85
	Not consuming the pills	50	105.22 ± 10.80	
	3 months	9	109.33 ± 10.46	
	4-23 months	18	102.44 ± 6.32	
<brow>-4</erow> Systolic blood pressure (mmHg)	24-36 months	23	110.73 ± 10.85	<brow>-4</erow> 0.04
	Not consuming the pills	50	71.04 ± 7.07	
	3 months	9	74.00 ± 8.29	
	4-23 months	18	71.11 ± 5.17	
<brow>-4</erow> Diastolic blood pressure (mmHg)	24-36 months	23	74.39 ± 8.28	<brow>-4</erow> 0.23
*ANOVA test				
BMI: Body mass index; WHR: Waist-hip ratio

**Table 3:** The mean and SD concerning the risk factors for increasing cardiovascular diseases amongst the users of the pills regarding the duration of the consumption of the pills


**Risk factors**	**The duration of the consumption of the pills**	**Numbers**	**Mean ± SD**	P-value*
	Not consuming the pills	50	3.28 ± 1.61	
	3 months	9	3.25 ± 0.96	
	4-23 months	18	2.83 ± 1.38	
<brow>-4</erow> Homocysteine levels (µmol/l)	24-36 months	23	4.87 ± 2.91	<brow>-4</erow> <0.001
	Not consuming the pills	50	159.74 ± 30.25	
	3 months	9	168.11 ± 25.12	
	4-23 months	18	180.16 ± 39.66	
<brow>-4</erow> Cholesterol (mg/dl)	24-36 months	23	186.04 ± 41.51	<brow>-4</erow> 0.01
	Not consuming the pills	50	84.84 ± 24.70	
	3 months	9	91.02 ± 22.68	
	4-23 months	18	102.51 ± 30.15	
<brow>-4</erow> LDL-c (mg/dl)	24-36 months	23	104.64 ± 33.88	<brow>-4</erow> 0.01
	Not consuming the pills	50	93.60 ± 44.01	
	3 months	9	101.00 ± 38.69	
	4-23 months	18	125.22 ± 44.32	
<brow>-4</erow> Triglyceride (mg/dl)	24-36 months	23	144.69 ± 49.65	<brow>-4</erow> <0.001
	Not consuming the pills	50	56.18 ± 8.91	
	3 months	9	56.88 ± 10.31	
	4-23 months	18	55.16 ± 7.01	
<brow>-4</erow> HDL-c (mg/dl)	24-36 months	23	57.30 ± 8.90	<brow>-4</erow> 0.88
	Not consuming the pills	50	162.65 ± 90.91	
	3 months	9	139.14 ± 80.47	
	4-23 months	18	184.45 ± 95.85	
<brow>-4</erow> Nitric oxide (µM)	24-36 months	23	195.45 ± 88.42	<brow>-4</erow> 0.31
*ANOVA test				
LDL-c: Low-density lipoproteins cholesterol; HDL-c: High-density lipoproteins cholestero

## 4. Discussion

The present study revealed that there was a relationship between the duration of the consumption of the pills and the incidence of some risk factors for increasing cardiovascular diseases like HCY, cholesterol, triglyceride, and LDL-c levels. According to the results, there were no remarkable differences amongst the participants concerning BMI, WHR, and diastolic blood pressure, while there was a significant difference in the systolic blood pressure amongst the groups. A study carried out by Emokpae indicated that there was a comprehensible difference in the increased blood pressure amongst the long-term users of those pills (25). This finding was compatible with our results. However, other studies accomplished in the literature expressed that there were no specific associations between the consumption of the pills, weight gain (26), and the systolic and diastolic blood pressures (25-28). Although weight gain has been reported as a side effect of OCPs, there is a still lack of evidence supporting this notion (23). The published studies in this regard also suggest that there is no relationship between gaining weight and long-term usage of the pills. For example, Pelkman and coworkers demonstrated that consuming the pills had no influence on nutritional intake and energy consumption and did not lead to weight gain (29).

The present study indicated that there was a comprehensible difference in the mean value of the plasma level of HCY due to the difference in the duration of the pill consumption. A study carried out by Fallah and colleagues on 100 women with/without a history of consuming the pills in Tehran, Iran, showed a result compatible with ours (12).

The results proved that although the mean values of NO and HDL-c levels among the women using the pills for the longest period were high, there were no significant differences in comparison with the other groups in this regard. According to other findings, the mean values of triglyceride, cholesterol, and LDL-c levels among the women consuming the pills for the longest time were high, which in this finding was comprehensible amongst the users of the pills statistically. Moreover, the study accomplished by Emokpae and colleagues to investigate the influence of long-term usage of the pills on lipid profiles showed results compatible with our results (25). Contrasting to this study, a study by Guazzelli to investigate lipid profiles revealed that the duration of the pill consumption made no statistically significant changes in this regard (30). Dissimilar to the conducted studies, examining the influence of OCPs for a short period of time, investigating the effects on women attending only one public health clinic, and also considering no difference between the prescribed pills, the current study has some strengths as follows: only LD contraceptive pills were used for a long time and they were given to the women who attended several public health clinics.

There were also some limitations in this study. First, the limitation of costs for this project due to which the study was conducted on a relatively small population. Therefore, some relationships were not detected due to the low sample size. Second, the study was performed in a limited time so that only one blood sample was taken from each participant regardless of the follicular and luteal phase of the menstrual cycle.

## 5. Conclusion

The results proposed that there was a statistically significant association concerning the incidence rate of risk factors for increasing cardiovascular diseases between the women who never used LD contraceptive pills and the counterparts consuming the pills for a long time. Hence, it can be concluded that consuming LD contraceptive pills containing low hormones for a long time may be accompanied by an increased risk of developing cardiovascular diseases. Therefore, given the significance of this obstacle, it is recommended that the obtained results of this study should be taken into account by the healthcare providers while prescribing the pills.

##  Conflict of Interests 

The authors declare that there is no conflicts of interests.

## References

[1] Kazemy T., Sharifzadeh Gh. (2004). Ten-year changes in mortality and risk factors in acute myocardial infarction in Birjand (1994-2003). Horizon Med Sci. *Sharifzadeh Gh. Ten-year changes in mortality and risk factors in acute myocardial infarction in Birjand (1994-2003). Horizon Med Sci*.

[2] Robbins C. L., Farr S. L., Zapata L. B., D’Angelo D. V., Callaghan W. M. (2015). Postpartum contraceptive use among women with a recent preterm birth. *American Journal of Obstetrics & Gynecology*.

[3] Alonso de Leciñana M., Egido J. A. (2006). Estrogens as Neuroprotectants against Ischemic Stroke. *Cerebrovascular Disease*.

[4] Ribeiro C. C., Shimo A. K., Lopes M. H., Lamas J. L. (2018). Effects of different hormonal contraceptives in women's blood pressure values. *Revista Brasileira de Enfermagem*.

[5] Sbarouni E., Kyriakides Z. S., Kremastinos D. T. (2005). The Effect of Hormone Replacement Therapy and Simvastatin on Plasma Homocysteine. *Journal of Women's Health*.

[6] Gupta M., Sharma P., Garg G., Kaur K., Bedi G. K., Vij A. (2005). Plasma homocysteine: an independent or an interactive risk factor for coronary artery disease. *Clinica Chimica Acta*.

[7] Vasan R. S. (2003). Plasma Homocysteine and Risk for Congestive Heart Failure in Adults Without Prior Myocardial Infarction. *Journal of the American Medical Association*.

[8] Kannel W. B. (2005). Overview of hemostatic factors involved in atherosclerotic cardiovascular disease. *Lipids*.

[9] Serapinas D., Boreikaite E., Bartkeviciute A., Bandzeviciene R., Silkunas M., Bartkeviciene D. (2017). The importance of folate, vitamins B6 and B12 for the lowering of homocysteine concentrations for patients with recurrent pregnancy loss and MTHFR mutations. *Reproductive Toxicology*.

[10] Ardawi M. S. M., Rouzi A. A., Qari M. H., Dahlawi F. M., Al-Raddadi R. M. (2002). Influence of age, sex, folate and vitamin B12 status on plasma homocysteine in Saudis. *Saudi Medical Journal*.

[11] Mirzaei N., Dehpour AR. (2005). Investigation of homocystein plasma level in cholestatic rat and its effect on nitric oxide secretion in liver. *Sci J Hamadan Univ Med Sci*.

[12] Merki-Feld G. S., Imthurn B., Keller P. J. (2002). Effects of two oral contraceptives on plasma levels of nitric oxide, homocysteine, and lipid metabolism. *Metabolism - Clinical and Experimental*.

[13] Merki-Feld G. S., Imthurn B., Keller P. J. (2002). Effects of two oral contraceptives on plasma levels of nitric oxide, homocysteine, and lipid metabolism. *Metabolism - Clinical and Experimental*.

[14] Mirershadi F., Faghihi M., Dehpour AR. (2009). Effect of endogenous nitric oxide on cardiac ischemic preconditioning in rat. *Repository of Research*.

[15] Lussana F., Zighetti M. L., Bucciarelli P., Cugno M., Cattaneo M. (2003). Blood levels of homocysteine, folate, vitamin B6 and B12 in women using oral contraceptives compared to non-users. *Thrombosis Research*.

[16] Rosendaal F., Helmerhorst F., Vandenbroucke J. (2002). Female Hormones and Thrombosis. *Arteriosclerosis, Thrombosis, and Vascular Biology*.

[17] Akbarzadeh M., SHarifi N. (2013). Comparison of cardiovascular disease in women with OCP use and without OCP use in hospitals of. *Iranian Journal of Nursing Research*.

[18] Tuchman E. (2015). Women's injection drug practices in their own words: A qualitative study. *Harm Reduction Journal*.

[19] Fallah S., Nouroozi V., Seifi M., Samadikuchaksaraei A., Aghdashi E. M. (2012). Influence of Oral Contraceptive Pills on Homocysteine and Nitric Oxide Levels: As Risk Factors for Cardiovascular Disease. *Journal of Clinical Laboratory Analysis*.

[20] Kumar A., Palfrey H. A., Pathak R., Kadowitz P. J., Gettys T. W., Murthy S. N. (2017). The metabolism and significance of homocysteine in nutrition and health. *Nutrition & Metabolism*.

[21] Eslami A., Lotfaliany M., Akbarpour S., Azizi F., Hadaegh F. (2018). Trend of cardiovascular risk factors in the older Iranian population: 2002–2014. *Geriatrics & Gerontology International*.

[22] Esmaillzadeh A., Mirmiran P., Azizi F. (2004). Waist-to-hip ratio is a better screening measure for cardiovascular risk factors than other anthropometric indicators in Tehranian adult men. *International Journal of Obesity*.

[23] Tohidi M., Assadi M., Dehghani Z., Vahdat K., Emami SR., Nabipour I. (2012). High sensitive C-reactive protein and ischemic heart disease, a population- based study. Iran South Med J. *High sensitive C-reactive protein and ischemic heart disease*.

[24] Saw S., Yuan J., Ong C., Arakawa K., Lee H., Coetzee G. A., Yu M. C. (2001). Genetic, dietary, and other lifestyle determinants of plasma homocysteine concentrations in middle-aged and older Chinese men and women in Singapore. *American Journal of Clinical Nutrition*.

[25] Lessner D. J., Lhu L., Wahal C. S., Ferry J. G. (2010). An engineered methanogenic pathway derived from the domains bacteria and archaea. *mBio*.

[26] Grossman D., Fuentes L. (2013). Over-the-counter access to oral contraceptives as a reproductive healthcare strategy. *Current Opinion in Obstetrics and Gynecology*.

[27] Azizi F., Ainy E., Mirmiran P., Habibian S. (2009). Contraceptive methods and risk factors of cardiovascular diseases in Tehranian women: Tehran Lipid and Glucose Study. *The European Journal of Contraception & Reproductive Health Care*.

[28] Lech MM., Ostrowska L. (2002). Effects of low-dose OCs on weight in women with central European nutritional habits and lifestyle. Contraception. *Ostrowska L. Effects of low-dose OCs on weight in women with central European nutritional habits and lifestyle. Contraception*.

[29] Pelkman C. L., Chow M., Heinbach R. A., Rolls B. J. (2001). Short-term effects of a progestational contraceptive drug on food intake, resting energy expenditure, and body weight in young women. *American Journal of Clinical Nutrition*.

[30] Guazzelli C. A., Lindsey P. C., de Araújo F. F., Barbieri M., Petta C. A., Aldrighi J. M. (2005). Evaluation of lipid profile in adolescents during long-term use of combined oral hormonal contraceptives. *Contraception*.

